# Biochemical control in intermediate- and high-risk prostate cancer after EBRT with and without brachytherapy boost

**DOI:** 10.1007/s00066-024-02245-3

**Published:** 2024-06-03

**Authors:** Matthias Moll, Łukasz Magrowski, Martina Mittlböck, Harald Heinzl, Christian Kirisits, Jakub Ciepał, Oliwia Masri, Gerd Heilemann, Rafał Stando, Tomasz Krzysztofiak, Gabriela Depowska, Andrea d’Amico, Tomasz Techmański, Anna Kozub, Wojciech Majewski, Rafał Suwiński, Piotr Wojcieszek, Jacek Sadowski, Joachim Widder, Gregor Goldner, Marcin Miszczyk

**Affiliations:** 1 Department of Radiation Oncology, Comprehensive Cancer Center, Medical University of Vienna, Währinger Gürtel 18–20, 1090 Vienna, Austria; 2 IIIrd, Maria Skłodowska-Curie National Research Institute of Oncology, Wybrzeże Armii Krajowej 15, 44-102 Gliwice, Poland; 3 Center for Medical Data Science, Medical University of Vienna, Vienna, Austria; 4 Radiotherapy Department, Holycross Cancer Centre, Kielce, Poland; 5 Brachytherapy Department, Maria Skłodowska-Curie National Research Institute of Oncology, Wybrzeże Armii Krajowej 15, 44-102 Gliwice, Poland; 6 Department of PET Diagnostic, Maria Sklodowska-Curie National Research Institute of Oncology Gliwice Branch, Wybrzeze Armii Krajowej 15, 44-101 Gliwice, Poland; 7 Radiotherapy Department, Maria Skłodowska-Curie National Research Institute of Oncology, Wybrzeże Armii Krajowej 15, 44-102 Gliwice, Poland; 8 IInd, Maria Skłodowska-Curie National Research Institute of Oncology, Wybrzeże Armii Krajowej 15, 44-102 Gliwice, Poland; 9 Department of Urology, Comprehensive Cancer Center, Medical University of Vienna, Währinger Gürtel 18–20, 1090 Vienna, Austria; 10 Collegium Medicum – Faculty of Medicine, WSB University, Dąbrowa Górnicza, Poland

## Abstract

**Purpose:**

External beam radiotherapy (EBRT) with or without brachytherapy boost (BTB) has not been compared in prospective studies using guideline-recommended radiation dose and recommended androgen-deprivation therapy (ADT). In this multicenter retrospective analysis, we compared modern-day EBRT with BTB in terms of biochemical control (BC) for intermediate-risk (IR) and high-risk (HR) prostate cancer.

**Methods:**

Patients were treated for primary IR or HR prostate cancer during 1999–2019 at three high-volume centers. Inclusion criteria were prescribed ≥ 76 Gy EQD2 (α/β = 1.5 Gy) for IR and ≥ 78 Gy EQD2 (α/β = 1.5 Gy) for HR as EBRT alone or with BTB. All HR patients received ADT and pelvic irradiation, which were optional in IR cases. BC between therapies was compared in survival analyses.

**Results:**

Of 2769 initial patients, 1176 met inclusion criteria: 468 HR (260 EBRT, 208 BTB) and 708 IR (539 EBRT, 169 BTB). Median follow-up was 49 and 51 months for HR and IR, respectively. BTB patients with ≥ 113 Gy EQD_2Gy_ experienced a stable, good BC outcome compared with BTB at lower doses. Patients treated with ≥ 113 Gy EQD_2Gy_ also experienced significantly improved BC compared with EBRT (10-year BC failure rates after ≥ 113 Gy BTB and EBRT: respectively 20.4 and 41.8% for HR and 7.5 and 20.8% for IR).

**Conclusions:**

In patients with IR and HR prostate cancer, BTB with ≥ 113 Gy EQD_2Gy_ offered a BC advantage compared with dose-escalated EBRT and lower BTB doses.

**Supplementary Information:**

The online version of this article (10.1007/s00066-024-02245-3) contains supplementary material, which is available to authorized users.

## Introduction

Prostate cancer (PC) is the most common non-skin cancer in men in the western world, accounting for almost 26% of all new cancer cases and 10.7% of all cancer deaths in the United States in 2021 [1]. Curative local treatment of the primary cancer includes surgery and radiotherapy [1]. For radiotherapy, treatment in unfavorable intermediate-risk and high-risk groups consists of external beam radiotherapy (EBRT) with androgen-deprivation therapy (ADT) with or without a brachytherapy boost (BTB) [1]. In randomized controlled trials, EBRT plus BTB has shown an advantage compared with EBRT alone [2–4]. From newer perspectives, however, these studies have some weaknesses. In the context of the current standard of care, patients in the EBRT arms received insufficient radiotherapy doses [2, 3] and no or insufficient prescription of ADT in cases of high-risk PC [3, 4]. The 12 months of ADT prescribed in the ASCENDE trial could be insufficient for patients with high-risk PC, and 18 months of ADT is as efficient as 36 months [5], whereas 6 months of ADT is not [6]. Furthermore, toxicities reported in the EBRT+BTB arm in the ASCENDE trial were considerably higher than for EBRT alone [7], as EBRT was not applied with modern intensity-modulated radiotherapy but with 3D conformal radiotherapy [4]. Using a current therapy schedule in the control arm, the FLAME trial showed a tumor control benefit after an EBRT boost to the tumor, demonstrating the benefits of further dose escalation [8].

In this study, we compared treatment with EBRT or BTB using modern therapy methods (dose, ADT, pelvic irradiation). Our aim was to determine if the benefits of BTB reported in earlier studies could be replicated using real-world data from a large cohort treated with modern-day EBRT.

## Materials and methods

This retrospective study comprised patients from one Austrian and two Polish tertiary oncologic centers. The study protocol was approved by the respective ethics review boards according to local laws and regulations in accordance with the Declaration of Helsinki. Ethics votes were EK 1678/2022 in Vienna and KB/430-08/22 in Gliwice.

Patients had to have been treated from January 1999 through December 2019. Inclusion criteria were treatment for primary PC; IR or HR PC according to the D’Amico classification [9]; receipt of conventionally fractionated or moderately hypofractionated EBRT with or without BTB; prescription of a total applied equivalent dose to the planning target volume (PTV) of ≥ 76 Gy EQD_2Gy_ for IR and ≥ 78 Gy EQD_2Gy_ for HR, assuming an α/β of 1.5 Gy; tumors staged as cN0/X and cM0; maximum prostate-specific antigen (PSA) level ≤ 50 µg/l; elective pelvic irradiation with total doses between 44 and 50.4 Gy in fractions of 1.8–2 Gy in HR patients and optional for IR cases; and administration of ADT in HR patients, also optional for IR cases.

For EBRT, the clinical target volume (CTV) included the prostate and the seminal vesicles. The CTV was expanded by 5–10 mm to create the PTV. The CTV for pelvic lymph node irradiation included the external, internal, and common iliac lymph nodes, up to the aortic bifurcation in Vienna (usually L4/5), as well as the para-prostatic and obturator nodes. The CTV was expanded by 3–10 mm to arrive at the PTV. Doses were prescribed to 95% of the PTV, according to International Commission on Radiation Units and Measurements reports 50, 62, and 83 [10–12]. Treatment was delivered as 3D conformal, intensity-modulated radiotherapy or volumetric-modulated arc therapy. All patients were treated in the supine position with a full bladder.

The BTB consisted of high dose–rate brachytherapy, administered in one or two fractions of 10–15 Gy each. Planning was performed using ultrasound and taking information from MRI and PSMA-PET-CT scans into account, if available. No image fusion was performed. The CTV included the prostate. For T3 tumors, the tumor bearing areas (infiltration of the seminal vesicles or extra-prostatic extensions) were included. There was no further PTV margin added. The planning aim for dose parameters was a D90 of 100–105% of the prescribed dose and additionally V100 > 95%, V150 < 35%, and V200 < 10%. The optimized plans for D90 were so close to the planning aim dose (initial prescription) that these nominal dose values were used for further total EQD2 calculations and analysis. No focal boosts were performed. Dose distribution was optimized to be as homogenous as possible around the whole gland. However, due to the needle spacing and placement around the urethra, as well as the nature of HDR-BT with its steep decline in dose close to the catheters, this was not fully achieved. Brachytherapy was usually applied in the first week of treatment before EBRT initiation and the week after EBRT completion, if two fractions were given. In rare cases, both fractions were applied before or after EBRT with a break of 2 weeks in between. In this way, total repair could be assumed in all cases between the two brachytherapy fractions. The overall treatment time was limited to 7 weeks in all cases. A detailed list of patient distribution by country and dose distribution can be found in supplement 1–3 and 13. Dose constraints for BTB can be found in supplement 14.

ADT was prescribed at the discretion of the attending urologist but was recommended for 6 months in patients with IR PC and for 1.5–3 years in patients with HR PC [13]. ADT duration was measured for the duration of administration or until the first recurrence.

PSA values were collected at each follow-up. Follow-up was scheduled at least once per year. Biochemical control (BC) was the primary endpoint, and biochemical failure was defined using the Phoenix criteria (nadir +2 ng/ml). Overall survival data were collected from the local population census.

### Statistical analyses

Frequencies and percentages were used to describe qualitative variables, and quantitative variables were described with medians and the lower (Q1) and upper quartiles (Q3). Association between calendar time of BTB and dose were depicted using a scatterplot and fitted by a locally estimated scatterplot smoothing (i.e., LOESS) curve with a smoothing parameter of 0.25. Median follow-up was computed with the inverse Kaplan–Meier method regarding disease-free survival (first occurrence of either BC failure or death were censoring events). Cumulative incidence functions were applied to estimate and graphically illustrate the incidence of cause-specific failures (BC, overall survival). Because of the small number of deaths as a first event, cumulative incidence functions and 1‑Kaplan–Meier estimates were close (see Supplementary Figures 11–12).

The univariable and multivariable influence of covariates on BC failure was assessed with Cox regression models, the effects are quantified with hazard ratios with corresponding 95% confidence intervals. In this regard, the time of ADT treatment since radiation (ADT-time) was included as a time-dependent covariate, and a potentially non-linear effect of dose in EQD2 was modeled with restricted cubic splines [14].

Statistical computations were performed with SAS 9.4 (SAS Institute Inc., Cary, NC, USA).

P values ≤ 0.05 were considered statistically significant. Note that no adjustment for multiple testing was performed as the goals of this retrospective study were exploratory rather than confirmatory.

## Results

In total, 1176 patients met the inclusion criteria and were classified into two main groups (HR and IR) according to the D’Amico classification [9]. Both groups were further separated by treatment method, either EBRT or EBRT plus BTB. For the HR group, we identified 468 patients, 260 who received EBRT and 208 who received BTB. All of these patients received ADT and pelvic lymph node irradiation. The IR group included 708 patients, 539 treated with EBRT and 169 with BTB. In this group, ADT and lymph node irradiation were given at the discretion of the attending physician. Patient characteristics are listed in Table 1. The median prescribed high dose–rate brachytherapy was 20 Gy in two fractions of 10 Gy each. A detailed list of patient distribution by country and treatment type, as well as administered doses, can be found in Supplements 1–3.

**Table 1:** Patient characteristics

	High-risk			Intermediate-risk		
	EBRT	BT boost	BT boost EQD2 ≥ 113 Gy	EBRT	BT boost	BT boost EQD2 ≥ 113 Gy
*N* (%)	260 (100)	208 (100)	128 (100)	539 (100)	169 (100)	98 (100)
*T category, n (%)*						
T1c/2a	132 (51)	80 (38)	32 (25)	455 (84)	138 (82)	83 (85)
T2b	22 (8)	20 (10)	12 (9)	84 (16)	31 (18)	15 (15)
T2c or higher	106 (41)	108 (52)	84 (66)	0 (0)	0 (0)	0 (0)
*ISUP grade, n (%)*						
1	20 (8)	106 (51)	55 (43)	148 (27)	102 (60)	54 (55)
2 or 3	46 (18)	56 (27)	36 (28)	391 (73)	67 (40)	44 (45)
4 or 5	194 (75)	46 (22)	37 (29)	0 (0)	0 (0)	0 (0)
Median initial PSA (IQR) in µg/l	11.8 (8.0/22)	22.4 (12.3/29.8)	20.0 (11.5/28.3)	10 (7/13.7)	12.7 (9.3/15.4)	12.5 (9.5/14.9)
Median age at therapy (IQR) in years	75 (71/78)	67 (64/72)	67 (65/73)	72 (68/76)	68 (63/74)	69 (63/74)
Pelvic irradiation, *n* (%)	260 (100)	208 (100)	128 (100)	182 (34)	94 (56)	47 (48)
ADT administered, *n* (%)	260 (100)	208 (100)	128 (100)	330 (61)	151 (89)	86 (88)
Median duration of ADT (IQR)	19 (9/36)	26 (15/34)	27 (18/35)	6 (0/19)	12.1 (5.7/23.2)	13 (6.1/23.8)
Median EQD_2Gy_ dose applied in Gy (IQR), α/β 1.5 Gy	82.1 (78/84.1)	115.7 (86.9/115.7)	115.7 (115.7/115.7)	78 (76/82.1)	115.7 (86.9/115.7)	115.7 (115.7/117.6)
Number of patients receiving at least 113 Gy EQD_2Gy_ (%)	0 (0%)	128 (62)	128 (100)	0 (0)	98 (58)	98 (100)
Median follow-up (IQR) in months	48 (24/60)	73 (44/106)	67 (43/95)	48 (32/72)	74 (34/110)	69 (33/104)
*Number of factors defining high risk *[9]*, n (%)*						
1	154 (59)	149 (72)	82 (64)	–	–	–
2	95 (37)	50 (24)	38 (30)	–	–	–
3	11 (4)	9 (4)	8 (6)	–	–	–
*Number of factors defining intermediate risk *[9]*, n (%)*						
1	–	–	–	357 (66)	121 (72)	68 (69)
2	–	–	–	159 (29)	45 (27)	28 (29)
3	–	–	–	23 (4)	3 (2)	2 (2)

*ADT* androgen-deprivation therapy; *BC* biochemical control; *BT* brachytherapy; *EBRT* external beam radiotherapy; *EQD*_*2Gy*_ equivalent dose in 2‑Gy fractions; *IQR* interquartile range

Risk definitions according to D’Amico et al. [9]: high risk, iPSA > 20 µg/l or cT2c or higher or Gleason score 8 or higher; intermediate risk, iPSA > 10 µg/l but ≤ 20 µg/l or cT2b or Gleason score 7

Percentages may not sum to 100% due to rounding errors

**Fig. 1:**
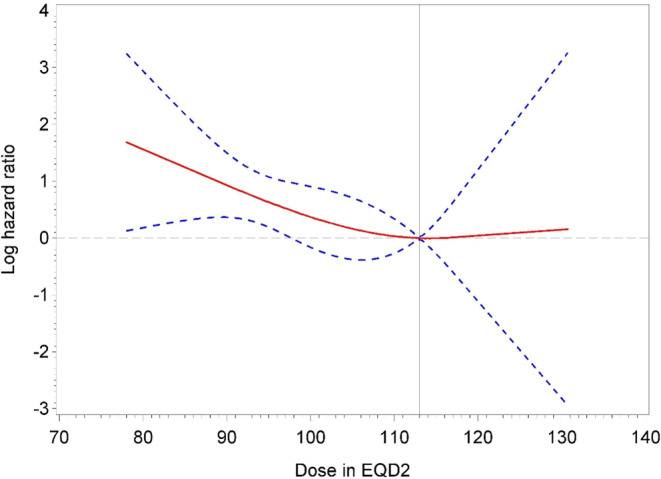
Patients with high-risk prostate cancer treated with brachytherapy boost. The log hazard ratio function (*red solid line*) and a corresponding 95% pointwise confidence band (*blue dashed lines*) were estimated by a restricted cubic spline to quantify the effect of dose in EQD2 on time to biochemical control failure. Note that smaller log hazard ratios indicate superior biochemical control. A reference value of 113 Gy *(grey vertical solid line*) was applied. The three spline knots were placed at 86, 115, and 118 Gy

**Fig. 2:**
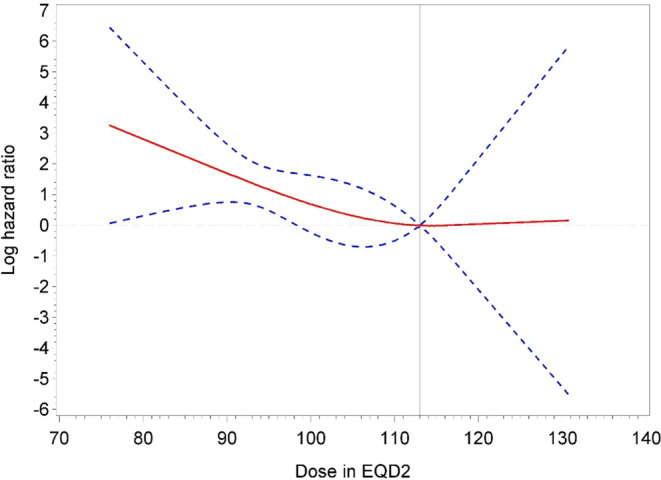
Patients with intermediate-risk prostate cancer treated with brachytherapy boost. The log hazard ratio function (*red solid line*) and a corresponding 95% pointwise confidence band (*blue dashed lines*) were estimated by a restricted cubic spline to quantify the effect of dose in EQD2 on time to biochemical control failure. Note that smaller log hazard ratios indicate superior biochemical control. A reference value of 113 Gy (*grey vertical solid line*) was applied. The three spline knots were placed at 87, 114, and 118 Gy

Because of the long time frame of inclusion, we performed an analysis comparing treatment time of BTB and prescribed dose, as shown in Supplement 4. Finding an increase in prescribed doses during that time, we fitted spline curves (Fig. 1 for HR and Fig. 2 for IR) for dose responses in BTB. The curves showed the lowest risks for BC failure at doses of 113 Gy EQD2 or higher, matching the dose recommendations of the GEC-ESTRO ACROP prostate brachytherapy guidelines [14]. We also adjusted dose effects for potential confounders (Supplements 5 and 6), and still found a similar dose-response effect. Of all patients receiving a BTB with < 113 Gy, 12 with HR and 7 with IR were treated after 2010. Thus, we focused on the comparison of patients with EBRT and BTB ≥ 113 Gy. Results for the comparison of EBRT and dose-independent BTB are shown in the supplements.

We compared both treatment modalities within each risk group in terms of BC. Overall, the number of BC failures in the HR group was 38 after EBRT (among 260 patients, 14.6%) and 40 (among 208 patients, 19.2%) in the BTB group. In the IR group, we observed 55 events (among 539 patients, 10.2%) after EBRT and 27 events (among 169 patients, 16.0%) after BTB.

A total of 128 patients (62%) in the HR BTB group received ≥ 113 Gy EQD2. We observed 14 events (11%) of BC failure in this group. For IR, 98 patients (58%) received ≥ 113 Gy EQD_2Gy_ in the BTB group. We observed 4 (4%) events of BC failure.

**Fig. 3:**
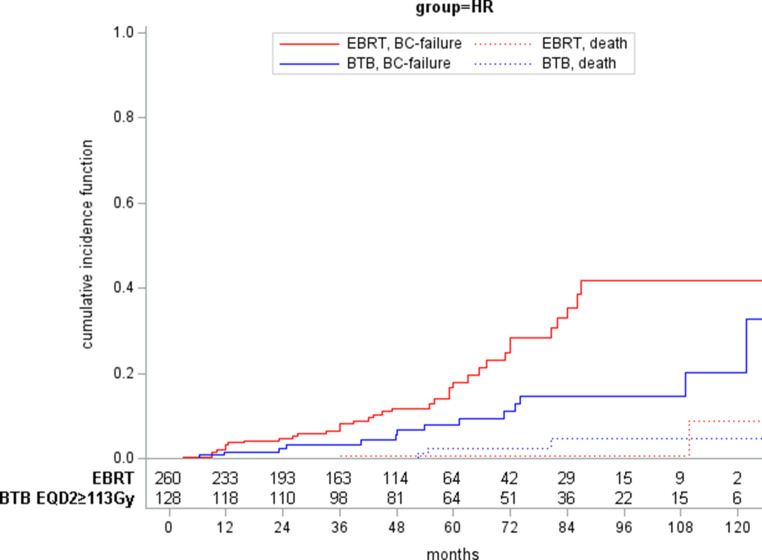
Cumulative incidence functions of patients with high-risk prostate cancer treated with either external beam radiotherapy (EBRT) or EBRT and an additional brachytherapy boost (BTB) of at least 113 Gy EDQ2

**Fig. 4:**
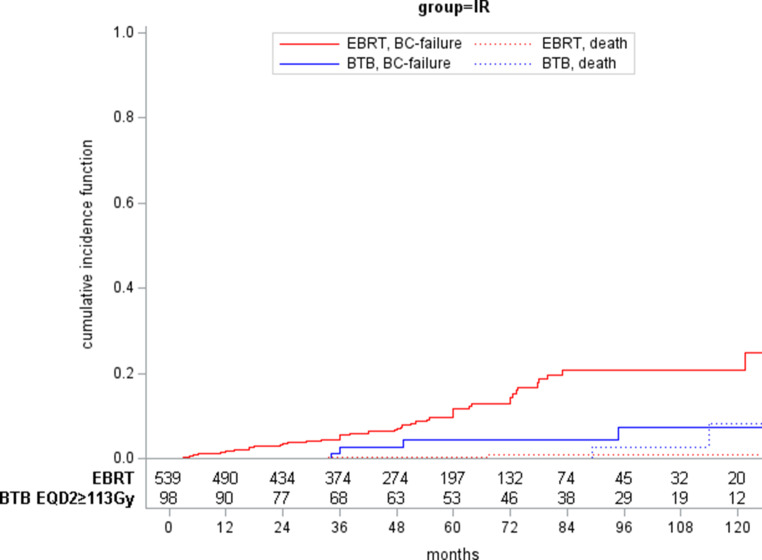
Cumulative incidence functions of patients with intermediate-risk prostate cancer treated with either external beam radiotherapy (EBRT) or EBRT and an additional brachytherapy boost (BTB) of at least 113 Gy EDQ2

Time to BC failure and death is shown by cumulative incidence functions in Figs. 3 and 4 for HR and IR, respectively. The 10-year BC failure rates were 20.4 and 41.8% for HR and 7.5 and 20.8% for IR after treatment with ≥ 113 Gy BTB and EBRT, respectively.

The results from univariable and multivariable analyses of patients receiving EBRT or BTB ≥ 113 Gy in terms of BC failure are shown in Table 2 for HR and Table 3 for IR. Two multivariable analyses were performed, one with baseline variables known before radiotherapy and the other that included ADT-time as a time-dependent covariable. For IR, the baseline variable ADT prescription was excluded for the second multivariable model because of the intrinsic association of ADT prescription and ADT duration. The same analyses were also performed for all BTB patients, with results given in Supplements 7–10.

**Table 2:** Results of univariable and multivariable analyses in terms of BC failure in patients with HR PC and brachytherapy doses of at least 113 Gy EQD2

		Hazard ratio	95% lower CI	95% upper CI	*P*
Univariable	ADT time (months)	0.9858	0.9656	1.0064	0.1766
	ISUP 2 or 3 vs 1	1.2311	0.5327	2.8455	0.6266^(7)^
	ISUP 4 or 5 vs 1	1.4933	0.7393	3.0165	0.2636^(1)^
	Initial PSA in ng/ml	1.0215	0.9979	1.0456	0.0746
	RT duration in days	1.0271	0.9977	1.0573	0.0713
	T category, T2b vs T1c/2a	0.9896	0.3243	3.0199	0.9853^(2)^
	T category, T2c/T3/T4 vs T1c/2a	1.7268	0.9233	3.2292	0.0872^(2)^
	**Age (years)**	**0.9548**	**0.9208**	**0.9901**	**0.0125**
	**Modality, EBRT vs BTB**	**2.4288**	**1.2956**	**4.5534**	**0.0056**
(1) -multivariable	ISUP 2 or 3 vs 1	0.9956	0.4198	2.3610	0.9921^(3)^
	ISUP 4 or 5 vs 1	2.1196	0.9424	4.7671	0.0693^(3)^
	**Initial PSA in ng/ml**	**1.0331**	**1.0062**	**1.0607**	**0.0155**
	RT duration (days)	1.0134	0.9782	1.0498	0.4601
	**T category, T2b vs T1c/2a**	0.7392	0.2294	2.3817	0.6126^**(4)**^
	**T category, T2c/T3/T4 vs T1c/2a**	1.9798	1.0318	3.7987	0.0400^**(4)**^
	**Age (years)**	**0.9376**	**0.9043**	**0.9722**	**0.0005**
	**Modality, EBRT vs BTB**	**2.9763**	**1.5104**	**5.8652**	**0.0016**
(2) multivariable with ADT-time	**ADT time (months)**	**0.9745**	**0.9532**	**0.9963**	**0.0221**
	**ISUP 2 or 3 vs 1**	0.9380	0.3960	2.2216	0.8842^(5)^
	**ISUP 4 or 5 vs 1**	2.3544	1.0439	5.3101	0.0391^(5)^
	**Initial PSA in ng/ml**	**1.0423**	**1.0139**	**1.0714**	**0.0033**
	RT duration (days)	1.0130	0.9782	1.0491	0.4688
	**T category, T2b vs T1c/2a**	0.8077	0.2496	2.6136	0.7216^**(6)**^
	**T category, T2c/T3/T4 vs T1c/2a**	2.2687	1.1713	4.3944	0.0152^**(6)**^
	**Age (years)**	**0.9364**	**0.9032**	**0.9709**	**0.0004**
	**Modality, EBRT vs BTB**	**3.1485**	**1.5951**	**6.2147**	**0.0009**

Significant findings (*p* ≤ 0.05) are highlighted in bold

Note: ADT time is time-dependent and starts with radiation

Overall tests for variables with > 2 groups are indicated by superscripted numerals, as follows: *(1) P* = 0.5205, *(2) P* = 0.1720, *(3) P* = 0.0814, *(4) P* = 0.0473, *(5) P* = 0.0309, *(6) P* = 0.0204

*ADT* androgen-deprivation therapy; *BTB* brachytherapy boost; *CI* confidence interval; *EBRT* external beam radiotherapy; *ISUP* International Society of Urological Pathology; *PSA* prostate-specific antigen; *RT* radiotherapy

**Table 3:** Univariable and multivariable analyses in terms of BC failure in patients with IR BC and brachytherapy doses of at least 113 Gy EQD2

		Hazard ratio	95% lower CI	95% upper CI	*P*
Univariable	**ADT time (months)**	**0.9709**	**0.9472**	**0.9951**	**0.0186**
	**ADT, yes vs no**	**0.5500**	**0.3284**	**0.9212**	**0.0231**
	ISUP 2 or 3 vs 1	1.6641	0.9245	2.9954	0.0894
	Pelvic RT, yes vs no	0.9912	0.5778	1.7004	0.9745
	Initial PSA in ng/ml	0.9831	0.9293	1.0400	0.5517
	RT duration (days)	1.0295	0.9994	1.0606	0.0552
	T category, T2b vs T1c/2a	0.6848	0.3240	1.4476	0.3215
	Age (years)	1.0227	0.9833	1.0638	0.2629
	**Modality, EBRT vs BTB**	**3.8148**	**1.3719**	**10.6072**	**0.0103**
1‑multivariable	ADT, yes/no	0.5983	0.3386	1.0571	0.0769
	ISUP 2 or 3 vs 1	1.4697	0.7390	2.9229	0.2724
	Pelvic RT, yes vs no	1.1309	0.5944	2.1514	0.7078
	Initial PSA in ng/ml	1.0158	0.9468	1.0898	0.6624
	RT duration (days)	1.0201	0.9835	1.0581	0.2856
	T category, T2b vs T1c/2a	0.7265	0.3363	1.5694	0.4161
	Age (years)	1.0051	0.9655	1.0463	0.8050
	Modality, EBRT vs BTB	2.7233	0.9286	7.9863	0.0680
2‑multivariable with ADT-time	**ADT time (months)**	**0.9690**	**0.9444**	**0.9943**	**0.0166**
	ISUP 2 or 3 vs 1	1.6131	0.8045	3.2344	0.1779
	Pelvic RT, yes vs no	1.1486	0.6079	2.1700	0.6696
	Initial PSA in ng/ml	1.0242	0.9549	1.0987	0.5032
	RT duration (days)	1.0185	0.9816	1.0568	0.3293
	T category, T2b vs T1c/2a	0.7474	0.3475	1.6074	0.4562
	Age (years)	0.9995	0.9604	1.0403	0.9813
	**Modality, EBRT vs BTB**	**2.9900**	**1.0134**	**8.8221**	**0.0472**

*ADT* androgen-deprivation therapy; *BTB* brachytherapy boost; *CI* confidence interval; *EBRT* external beam radiotherapy; *ISUP* International Society of Urological Pathology; *PSA* prostate-specific antigen; *RT* radiotherapy

Significant findings (*p* ≤ 0.05) are highlighted in bold

Note: ADT time is time-dependent and starts with radiation

## Discussion

Currently, three major prospective randomized trials have investigated the role of BTB compared with EBRT alone, mostly in patients with IR and HR PC [2–4]. Each trial showed an advantage for BTB regarding BC, but none showed significant improvement for overall survival after BTB [2, 15, 16]. In two of these studies, EBRT groups received insufficient radiation doses by current guidelines [2, 3], and IR and HR PC were included without distinction. Moreover, ADT was not used at all in one study [3], and in another, it was used for an insufficient duration for HR PC [4]. Only one study included pelvic irradiation [4], a factor that also was associated with improvement in BC for HR PC in the POP-RT trial [17], and in IR PC [18]. In the current study, we investigated total dose and the eventual influence of BTB on BC based on real-world data and with up-to-date treatment standards, stratifying for IR and HR PC.

In the univariable and multivariable analyses, dose strongly influenced BC in both IR and HR PC. Even with current standards in EBRT, such as sufficient ADT combined with pelvic irradiation in HR and an increased EBRT dose, the benefit of further dose escalation using BTB with a dose of at least 113 Gy EQD2 persisted.

We found a significant influence of ADT duration on BC in both the IR and HR groups. This finding is important because of a tendency in patterns of care to reduce ADT when dose escalation is employed [19, 20], despite studies showing the importance of ADT in combination with BTB in IR [21] and HR [22] PC. In their retrospective study, Kishan et al. also found improvements in disease-specific survival when comparing BTB with ADT and EBRT with ADT, but many patients with EBRT and ADT received insufficient EBRT doses [23]. A propensity score–matched analysis by Tamiharda et al. showed no significant difference between EBRT and BTB, although many patients did not receive ADT or pelvic irradiation in that study, and the BTB dose was only 100 Gy EQD_2Gy_ [24].

In considering toxicity, most [7, 25] but not all [2] studies showed increased toxicity with BTB. This consideration is relevant because the FLAME trial, by providing dose escalation as a simultaneously integrated boost, showed improved BC without increasing toxicity or impacting quality of life [8]. The results offer a potential alternative to BTB if further dose escalation is desired, but the trial did not show a benefit regarding overall survival with a median follow-up of 72 months. In a propensity score–matched analysis, we also found a survival benefit of dose escalation with BTB after a median follow-up of 117.8 months [26], leaving open the possibility that this outcome could still change in the FLAME trial.

The use of ultrasound-based dose planning for BTB is accepted state of the art. Using a 0-mm margin for the PTV is important. Comparing dose values with series applying a margin of 3–5 mm might result in uncertainties, as margins even compensated with more interstitial needles for conformal plans still result in higher CTV D90 doses if the planning aim is extended to the D90 for a larger PTV. The reported results for prescribed doses < 113 Gy EQD2 might be compared to series applying a D90 to a PTV with margins of much lower doses than this threshold.

The calculation of the EQD2 is limited to the cases involving use of BTB. For this reason, the spatial dose distribution of BTB cases cannot be directly compared with external beam dose applying the EQD2 concept. Total BTB dose distributions reported using a D90 include large parts of the prostate treated at substantially higher doses, especially within the peripheral zone, where interstitial needles usually are placed. The entire EQD2 comparison also depends on the contribution of EBRT versus BTB doses, which changes the entire total spatial dose distribution between the homogeneous EBRT and heterogeneous brachytherapy portion. The heterogeneity was caused by needle spacing and placement around the urethra, as well as the sharp decline in dose around the inserted catheters. Most cases included in this comparison involved 50–54 Gy EBRT, and the total EQD2 difference was dominated by 10 Gy versus 15 Gy applied in one or two fractions. Uncertainties included in the simple EQD2 summation are therefore limited.

This study has some further limitations, however, most prominently its retrospective nature. This feature could have led to a bias through informative censoring due to center effects in follow-up and might explain the BC differences in the HR group, as shown in Fig. 1. In the HR group in particular, some characteristics (e.g., age, IR, ADT use and duration and median follow-up) differed between treatment arms, which would not have been the case in a randomized controlled trial and is especially problematic for ADT use and duration as well as the short follow-up, especially in the EBRT group. We tried to mitigate this limitation by applying multivariable analyses, which confirmed our results showing that dose escalation led to improved BC. Another limitation is the uneven distribution of treatment arms between countries, as shown in supplement 1, as well as the long time frame of 20 years. For example, a recent study by Michalski et al. showed no benefit regarding tumor control of EBRT with BTB compared to brachytherapy alone [27].

Nevertheless, we report an analysis of data from a large group of patients treated according to current EBRT standards compared with high dose–rate BTB and provide evidence supporting the dose recommendation of 113–121 Gy EQD_2Gy_, in keeping with the GEC-ESTRO ACROP prostate brachytherapy guidelines [14]. The findings show a clear dose-response curve for both IR and HR PC.

## Conclusions

BTB at ≥ 113 Gy EQD_2Gy_ offers benefit for patients with IR or HR PC when compared with current EBRT and BTB at < 113 Gy EQD_2Gy_. We found a dose effect for both IR and HR PC. However, the aforementioned limitations, especially regarding ADT use and the short median follow-up, apply.

## Supplementary Information

The supplements include detailed distributions of patients and prescribed doses, further log hazard ratio functions, cumulative incidence functions and uni- and multivariable analyses.

## Data Availability

Data are available upon reasonable request for confirmed researchers who request it from the authors.
