# Supplementary material for: Biochemical control in intermediate- and high-risk prostate cancer after EBRT with and without brachytherapy boost
Source: Strahlenther Onkol. 2024 Jun 3;201(1):11–9. doi: 10.1007/s00066-024-02245-3 (PMC11739258; doi:10.1007/s00066-024-02245-3)
Supplement: Supplementary file 1 — The supplements include detailed distributions of patients and prescribed doses, further log hazard ratio functions, cumulative incidence functions and uni- and multivariable analyses. [file 66_2024_2245_MOESM1_ESM.docx]

Supplement 1. Distribution of patients by country of treatment, risk group, and treatment type.

|  | Poland | Austria | Total |
| --- | --- | --- | --- |
| High risk | 238 | 230 | 468 |
| EBRT | 30 | 230 | 260 |
| BT Boost | 208 | 0 | 208 |
| Intermediate risk | 384 | 324 | 708 |
| EBRT | 215 | 324 | 539 |
| BT Boost | 169 | 0 | 169 |
| Total for high risk and intermediate risk | 622 | 554 | 1176 |

Supplement 2. Dose distribution for the high-risk group; if dose per fraction is not 1.8–2 Gy, the dose per fraction is mentioned.

| Fractionation | EBRT (number of patients) | BTB (number of patients) |  | Dose (EQD_2Gy_, α/β 1.5 Gy) in Gy |  |
| --- | --- | --- | --- | --- | --- |
| 78 Gy EBRT/44–50.4 Gy Pelvic | 123 | - |  | 78/44–50.4 |  |
| 73 Gy (2.4–2.6 Gy per fraction)/50 Gy Pelvic | 10 | - |  | 82/50 |  |
| 74 Gy (2.4–2.8 Gy per fraction)/50 Gy Pelvic | 127 | - |  | 84/50 |  |
| 46 Gy EBRT/46 Gy Pelvic+10.5 Gy BTB | - | 1 |  | 82/46 |  |
| 46 Gy EBRT/44–46 Gy Pelvic+20 Gy BTB | - | 4 |  | 112/44–46 |  |
| 46 Gy EBRT/44–46 Gy Pelvic+21 Gy BTB | - | 9 |  | 118 |  |
| 50 Gy EBRT/50 Gy Pelvic+10 Gy BTB |  | 1 |  | 83/50 |  |
| 50 Gy EBRT/44 Gy Pelvic+14 Gy BTB |  | 1 |  | 112/50 |  |
| 50 (.4) Gy EBRT/50 (.4) Gy Pelvic+20 Gy BTB | - | 117 |  | 116 (117)/50 (.4) |  |
| 54 Gy EBRT/44–46 Gy Pelvic+10 Gy BTB | - | 58 |  | 87/44–46 |  |
| 56 Gy EBRT/44 Gy Pelvic+10.5 Gy BTB |  | 1 |  | 92/44 |  |
| 60 Gy EBRT/50 Gy Pelvic+10 Gy BTB | - | 7 |  | 93/50 |  |
| 60 Gy EBRT/50 Gy Pelvic+11 Gy BTB | - | 1 |  | 99/50 |  |
| 60 Gy EBRT/50 Gy Pelvic+12 Gy BTB | - | 1 |  | 106/50 |  |
| 60 Gy EBRT/50 Gy Pelvic+15 Gy BTB | - | 2 |  | 131/50 |  |
| 60 Gy EBRT/50 Gy Pelvic+20 Gy | - | 1 |  | 126/50 |  |
| 64 Gy EBRT/44 Gy Pelvic+10.5 Gy BTB | - | 4 |  | 100/44 |  |

Supplement 3. Dose distribution intermediate risk; if dose per fraction is not 1.8–2.1 Gy, the dose per fraction is mentioned.

| Fractionation | EBRT (number of patients) | BTB (number of patients) | Dose (EQD_2Gy_, α/β 1.5 Gy) in Gy |  |  |
| --- | --- | --- | --- | --- | --- |
| 76 Gy EBRT | 163 | - | 76 |  |  |
| 76(.4) Gy EBRT/44–50(.4) Gy Pelvic | 70 | - | 76/44–50 (.4) |  |  |
| 73 Gy (2.4–2.6 Gy per fraction | 100 | - | 82 |  |  |
| 73 Gy (2.4–2.6 Gy per fraction)/45–50 Gy Pelvic | 77 | - | 82/45–50 Gy |  |  |
| 78 Gy EBRT | 94 | - | 78 |  |  |
| 78 Gy EBRT/44–50(.4) Gy Pelvic | 35 | - | 78/44–50(.4) |  |  |
| 46 Gy EBRT+20 Gy BTB | - | 3 | 112 |  |  |
| 46 Gy EBRT+21 Gy BTB | - | 11 | 118 |  |  |
| 46 Gy EBRT/44–46 Gy Pelvic+20 Gy BTB |  | 2 | 112/44–46 |  |  |
| 46 Gy EBRT/44–46 Gy Pelvic+21 Gy BTB | - | 10 | 118/44–46 |  |  |
| 48 Gy EBRT/48 Gy Pelvic+20 Gy BTB | - | 1 | 114 |  |  |
| 50 (.4) Gy EBRT+20 Gy BTB | - | 39 | 116 (117) |  |  |
| 50 (.4) Gy EBRT/44–50 (.4) Gy Pelvic+20 Gy BTB | - | 35 | 116 (117)/ 44–50 (.4) |  |  |
| 50 Gy EBRT/50 Gy Pelvic+21 Gy BTB | - | 1 | 122 |  |  |
| 54 Gy EBRT+10 Gy BTB | - | 12 | 87 |  |  |
| 54 Gy EBRT+10.5 Gy BTB | - | 4 | 90 |  |  |
| 54 Gy EBRT/44 Gy Pelvic+ 10 Gy BTB | - | 39 | 87/44 |  |  |
| 54 Gy EBRT/44 Gy Pelvic+ 10.5 Gy BTB | - | 2 | 90/44 |  |  |
| 60 Gy EBRT+10 Gy BTB | - | 5 | 93 |  |  |
| 60 Gy EBRT+15 Gy BTB | - | 1 | 130 |  |  |
| 60 Gy EBRT/44–50 Gy Pelvic+10 Gy BTB | - | 4 | 93/44–50 |  |  |


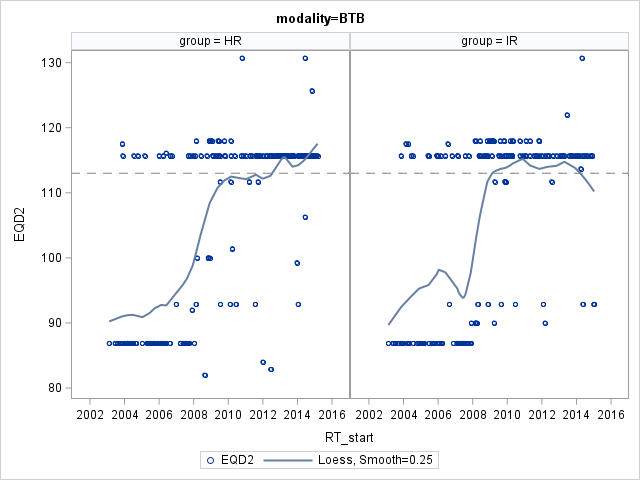


Supplement 4. Dose distribution of patients receiving brachytherapy boost (BTB) in the high-risk (HR, left side) and intermediate-risk (IR, right side) group, by year of treatment; the smoothing line was estimated by LOESS.


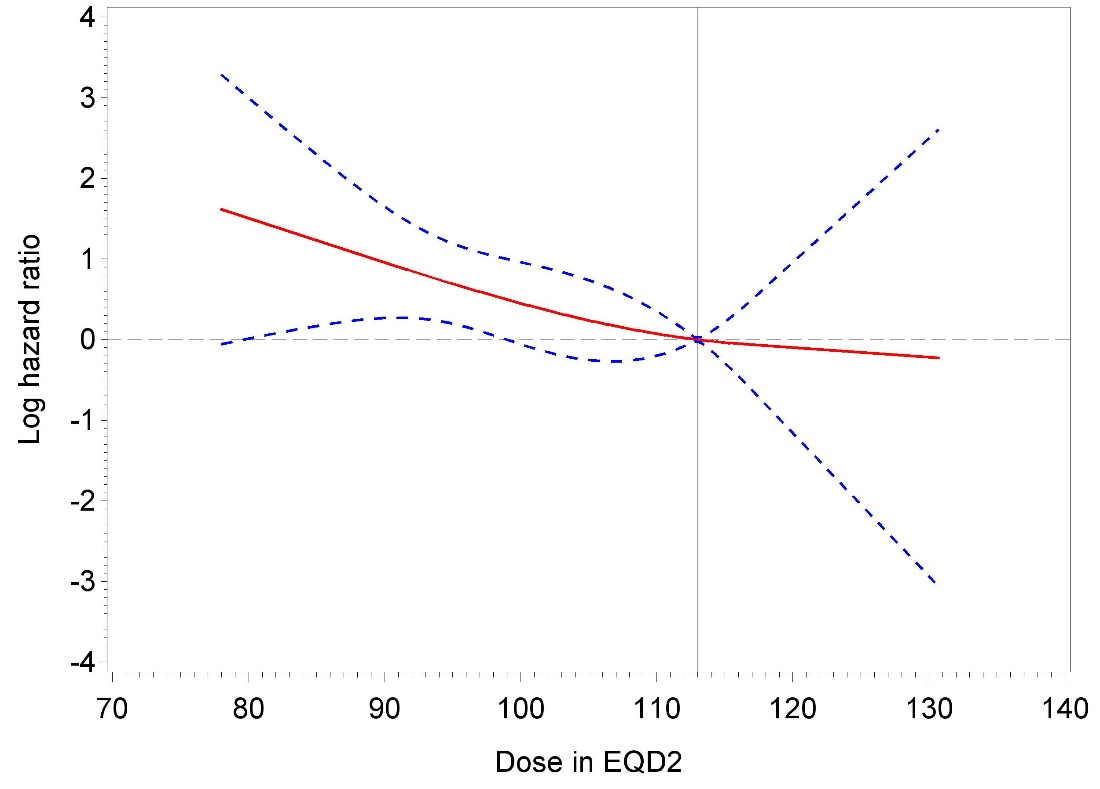


Supplement 5. Patients with high-risk prostate cancer treated with brachytherapy boost. The log hazard ratio function (red solid line) and a corresponding 95% pointwise confidence band (blue dashed lines) were estimated by a restricted cubic spline to quantify the effect of dose in EQD2 on time to biochemical control failure. Note that smaller log hazard ratios indicate superior biochemical control. A reference value of 113 Gy (grey vertical solid line) was applied. The three spline knots were placed at 86, 115, and 118 Gy. The estimated log hazard ratio function was adjusted for the covariates initial PSA, age, T category, and ISUP grade.


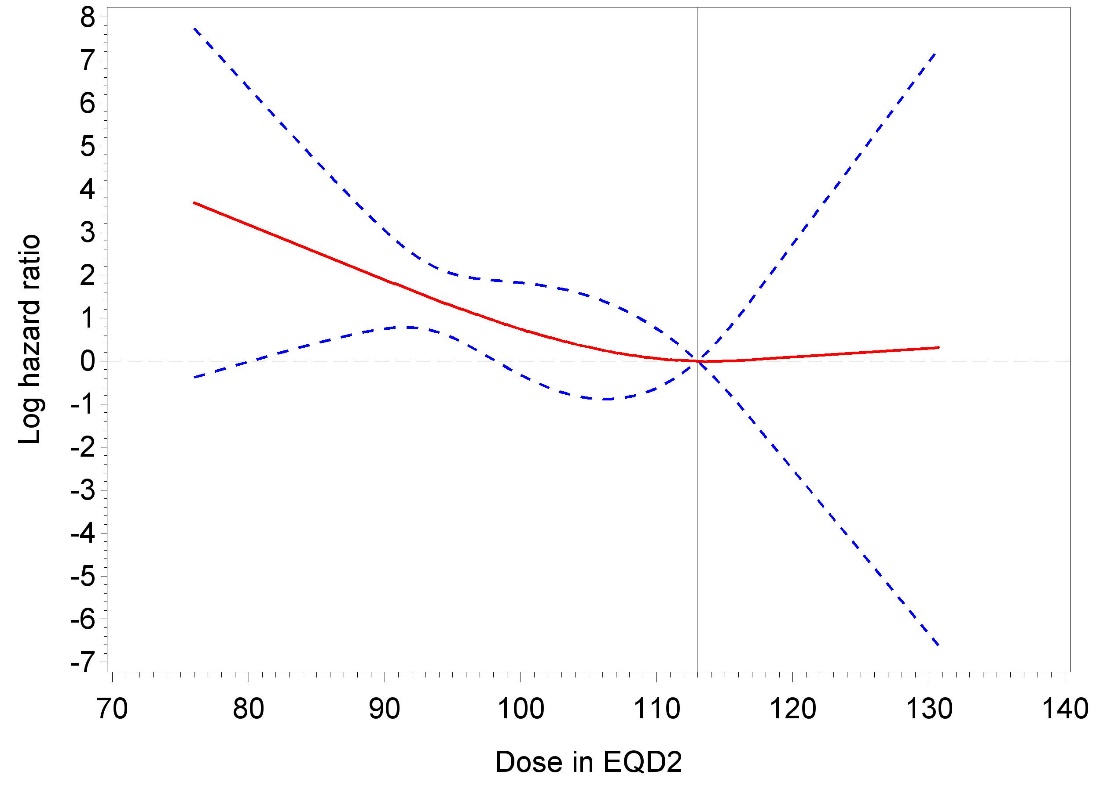


Supplement 6. Patients with intermediate-risk prostate cancer treated with brachytherapy boost. The log hazard ratio function (red solid line) and a corresponding 95% pointwise confidence band (blue dashed lines) were estimated by a restricted cubic spline to quantify the effect of dose in EQD2 on time to biochemical control failure. Note that smaller log hazard ratios indicate superior biochemical control. A reference value of 113 Gy (grey vertical solid line) was applied. The three spline knots were placed at 87, 114, and 118 Gy. The estimated log hazard ratio function was adjusted for the covariates initial PSA, T category, ISUP grade, application of ADT, pelvic irradiation, and age.


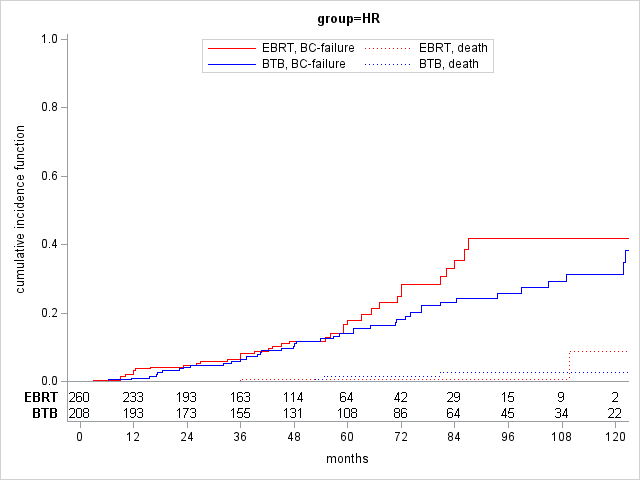


Supplement 7. Cumulative incidence functions of patients with high-risk (HR) prostate cancer treated with either external beam radiotherapy (EBRT) or EBRT and an additional brachytherapy boost (BTB). 10-year biochemical control (BC) failures were 31.3% and 41.8% after treatment with BTB and EBRT, respectively.


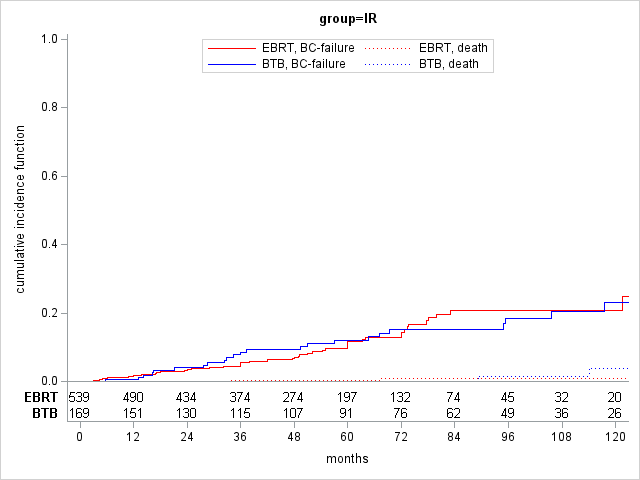


Supplement 8. Cumulative incidence functions of patients with intermediate-risk (IR) prostate cancer treated with either external beam radiotherapy (EBRT) or EBRT and an additional brachytherapy boost (BTB). 10-year biochemical control (BC) failures were 23.1% and 20.8% after treatment with BTB and EBRT, respectively.

Supplement 9. Univariable and multivariable analyses regarding biochemical control failure in patients with high-risk prostate cancer.

|  | | **HR** | **95% lower CI** | **95% upper CI** | **P** |
| --- | --- | --- | --- | --- | --- |
| **Univariable** | **ADT-time (months)** | 0.9861 | 0.9699 | 1.0024 | 0.0947 |
|  | **ISUP 2+3 vs 1** | 0.9296 | 0.5018 | 1.7222 | 0.8166^(1)^ |
|  | **ISUP 4+5 vs 1** | 1.1597 | 0.6926 | 1.9417 | 0.5732^(1)^ |
|  | **Initial PSA in ng/ml** | 1.0258 | 1.0070 | 1.0449 | 0.0068 |
|  | **RT duration (days)** | 1.0120 | 0.9880 | 1.0367 | 0.3293 |
|  | **T category, T2b vs T1c/2a** | 0.7045 | 0.2945 | 1.6853 | 0.4312^(2)^ |
|  | **T category, T2c/T3/T4 vs T1c/2a** | 0.9930 | 0.6235 | 1.5816 | 0.9765^(2)^ |
|  | **Age (years)** | 0.9576 | 0.9289 | 0.9871 | 0.0051 |
|  | **Modality, EBRT vs BTB** | 1.4185 | 0.8956 | 2.2467 | 0.1363 |
| **(1) multivariable** | **ISUP 2+3 vs 1** | 1.0781 | 0.5551 | 2.0941 | 0.8242^(3)^ |
|  | **ISUP 4+5 vs 1** | 1.7782 | 0.9372 | 3.3739 | 0.0782^(3)^ |
|  | **Initial PSA in ng/ml** | 1.0317 | 1.0104 | 1.0536 | 0.0035 |
|  | **RT duration (days)** | 1.0052 | 0.9779 | 1.0332 | 0.7120 |
|  | **T category, T2b vs T1c/2a** | 0.5461 | 0.2245 | 1.3283 | 0.1822 ^(4)^ |
|  | **T category, T2c/T3/T4 vs T1c/2a** | 1.1188 | 0.6870 | 1.8222 | 0.6518 ^(4)^ |
|  | **Age (years)** | 0.9439 | 0.9136 | 0.9753 | 0.0006 |
|  | **Modality, EBRT vs BTB** | 1.7738 | 1.0215 | 3.0804 | 0.0418 |
| **(2) multivariable with ADT-time** | **ADT-time (months)** | 0.9796 | 0.9622 | 0.9974 | 0.0247 |
|  | **ISUP 2+3 vs 1** | 1.0065 | 0.5179 | 1.9561 | 0.9847^(5)^ |
|  | **ISUP 4+5 vs 1** | 1.9000 | 0.9968 | 3.6218 | 0.0512^(5)^ |
|  | **Initial PSA in ng/ml** | 1.0377 | 1.0155 | 1.0604 | 0.0008 |
|  | **RT duration (days)** | 1.0074 | 0.9801 | 1.0354 | 0.6006 |
|  | **T category, T2b vs T1c/2a** | 0.5938 | 0.2428 | 1.4520 | 0.2533^(6)^ |
|  | **T category, T2c/T3/T4 vs T1c/2a** | 1.2296 | 0.7524 | 2.0095 | 0.4095^(6)^ |
|  | **Age (years)** | 0.9453 | 0.9150 | 0.9766 | 0.0007 |
|  | **Modality, EBRT vs BTB** | 1.8221 | 1.0458 | 3.1748 | 0.0342 |

ADT: androgen-deprivation therapy; BTB: brachytherapy boost; CI: confidence interval; EBRT: external beam radiotherapy; HR: hazard ratio; ISUP: International Society of Urological Pathology; PSA: prostate-specific antigen; RT: radiotherapy Significant findings (p≤0.05) are highlighted in red.

Note: ADT time is time-dependent and starts with radiation.

Overall test for variables with >2 groups are as follows:

(1) P=0.7362

(2) P=0.7175

(3) P=0.1543

(4) P=0.2881

(5) P=0.0781

(6) P=0.2549

Supplement 10. Univariable and multivariable analyses regarding biochemical control failure in patients with intermediate-risk prostate cancer.

|  | | **HR** | **95% lower CI** | **95% upper CI** | **P** |
| --- | --- | --- | --- | --- | --- |
| **Univariable** | **ADT time (months)** | 0.9662 | 0.9447 | 0.9882 | 0.0028 |
|  | **ADT, yes vs no** | 0.6579 | 0.4163 | 1.0396 | 0.0729 |
|  | **ISUP 2+3 vs 1** | 1.0066 | 0.6452 | 1.5703 | 0.9768 |
|  | **Pelvic RT, yes vs no** | 1.1091 | 0.7102 | 1.7320 | 0.6488 |
|  | **Initial PSA in ng/ml** | 1.0116 | 0.9654 | 1.0601 | 0.6278 |
|  | **RT duration (days)** | 1.0030 | 0.9781 | 1.0286 | 0.8133 |
|  | **T category, T2b vs T1c/2a** | 0.7053 | 0.3810 | 1.3058 | 0.2666 |
|  | **Age (years)** | 0.9985 | 0.9668 | 1.0313 | 0.9283 |
|  | **Modality, EBRT vs BTB** | 1.0814 | 0.6602 | 1.7711 | 0.7560 |
| **(1) multivariable baseline only** | **ADT, yes vs no** | 0.5837 | 0.3456 | 0.9860 | 0.0441 |
|  | **ISUP 2+3 vs 1** | 1.0173 | 0.5886 | 1.7581 | 0.9511 |
|  | **Pelvic RT, yes vs no** | 1.1809 | 0.7060 | 1.9751 | 0.5264 |
|  | **Initial PSA in ng/ml** | 1.0236 | 0.9657 | 1.0849 | 0.4330 |
|  | **RT duration (days)** | 1.0035 | 0.9752 | 1.0325 | 0.8128 |
|  | **T category, T2b vs T1c/2a** | 0.7416 | 0.3951 | 1.3918 | 0.3520 |
|  | **Age (years)** | 0.9938 | 0.9614 | 1.0274 | 0.7151 |
|  | **Modality, EBRT vs BTB** | 0.9825 | 0.5280 | 1.8282 | 0.9555 |
| **(2) multivariable with ADT-time** | **ADT time (months)** | 0.9590 | 0.9352 | 0.9834 | 0.0011 |
|  | **ISUP 2+3 vs 1** | 1.1988 | 0.6813 | 2.1093 | 0.5294 |
|  | **Pelvic RT, yes vs no** | 1.1933 | 0.7180 | 1.9831 | 0.4954 |
|  | **Initial PSA in ng/ml** | 1.0393 | 0.9804 | 1.1017 | 0.1956 |
|  | **RT duration (days)** | 1.0051 | 0.9765 | 1.0345 | 0.7320 |
|  | **T category, T2b vs T1c/2a** | 0.7950 | 0.4237 | 1.4916 | 0.4749 |
|  | **Age (years)** | 0.9877 | 0.9554 | 1.0210 | 0.4646 |
|  | **Modality, EBRT vs BTB** | 1.0413 | 0.5610 | 1.9329 | 0.8979 |

ADT: androgen-deprivation therapy; BTB: brachytherapy boost; CI: confidence interval; EBRT: external beam radiotherapy; HR: hazard ratio; ISUP: International Society of Urological Pathology; PSA: prostate-specific antigen; RT: radiotherapy.

Significant findings (p≤0.05) are highlighted in red.

Note: ADT time is time-dependent and starts with radiation.


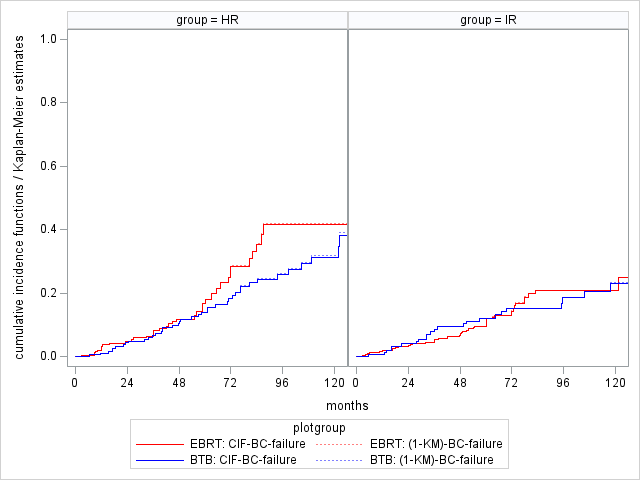


Supplement 11. Cumulative incidence functions (solid lines) and 1-Kaplan–Meier estimates (dotted lines) for biochemical control (BC) failure for EBRT and BTB in high risk and intermediate risk are in close agreement.


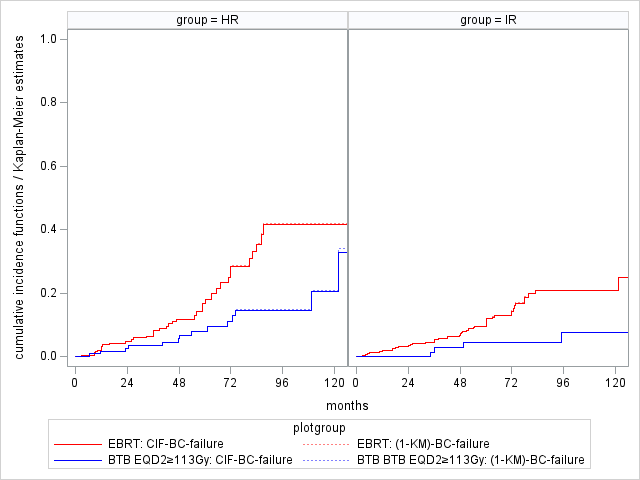


Supplement 12. Cumulative incidence functions (solid lines) and 1-Kaplan–Meier estimates (dotted lines) for biochemical control (BC) failure for EBRT and BTB with ≥113 Gy are in close agreement.


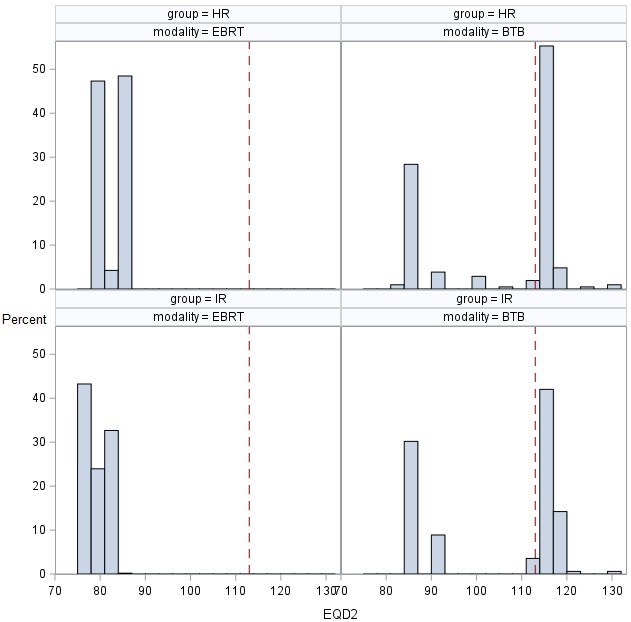


Supplement 13. Distribution of doses in Gy by treatment type and risk group

Supplement 14: Dose constraints for Brachytherapy boost

|  |  | Planning aims | Tolerated values |
| --- | --- | --- | --- |
| PTV | V100% | ≥ 95% | > 90%-95% |
|  | D90% | 100-115% pd | 100-104.9% pd |
|  | V150% | < 35% | 35.1-45% |
|  | V200% | < 12% | 12.1-17% |
| Urethra | D10% | < 120% pd | 120-133% pd |
|  | Dmax | < 130% pd | 130-140% pd |
| Bladder | V75% | ≤ 1 cm³ | 1-3 cm³ |
|  | Dmax | ≤ 100% pd | 100-113% pd |
| Rectum | V75% | ≤ 1 cm³ | 1-3 cm³ |
|  | Dmax | ≤ 100% pd | 100-113% pd |

Pd: prescribed dose
